# *Candida auris* Clinical Isolates Associated with Outbreak in Neonatal Unit of Tertiary Academic Hospital, South Africa

**DOI:** 10.3201/eid2910.230181

**Published:** 2023-10

**Authors:** Dikeledi Kekana, Serisha D. Naicker, Liliwe Shuping, Sithembiso Velaphi, Firdose L. Nakwa, Jeannette Wadula, Nelesh P. Govender, for GERMS-SA

**Affiliations:** National Institute for Communicable Diseases, Johannesburg, South Africa (D. Kekana, S.D. Naicker, L. Shuping, N.P. Govender);; University of the Witwatersrand, Johannesburg (D. Kekana, S. Velaphi, F.L. Nakwa, J. Wadula, N.P. Govender);; Chris Hani Baragwanath Academic Hospital, Johannesburg (S. Velaphi, F.L. Nakwa, J. Wadula);; University of Cape Town, Cape Town, South Africa (N.P. Govender);; University of Exeter, Exeter, United Kingdom (N.P. Govender)

**Keywords:** *Candida auris*, antifungal resistance, genome analysis, population genetics, fungi, antimicrobial resistance, South Africa

## Abstract

*Candida auris* was first detected at a university-affiliated hospital in Johannesburg, South Africa, in 2009. We used whole-genome sequencing to describe the molecular epidemiology of *C. auris* in the same hospital during 2016–2020; the neonatal unit had a persistent outbreak beginning in June 2019. Of 287 cases with culture-confirmed *C. auris* infection identified through laboratory surveillance, 207 (72%) had viable isolates and 188 (66%) were processed for whole-genome sequencing. Clade III (118/188, 63%) and IV (70/188, 37%) isolates co-circulated in the hospital. All 181/188 isolates that had a fluconazole MIC >32 µg/mL had *ERG11* mutations; clade III isolates had VF125AL substitutions, and clade IV isolates had K177R/N335S/E343D substitutions. Dominated by clade III, the neonatal unit outbreak accounted for 32% (91/287) of all cases during the study period. The outbreak may have originated through transmission from infected or colonized patients, colonized healthcare workers, or contaminated equipment/environment.

*Candida auris* is a multidrug-resistant yeast that causes invasive infections with high associated mortality in acute and long-term healthcare facilities worldwide (*1*,*2*). Although *C. auris* was initially described in 2009 in Japan, the earliest cases of infection in South Africa were reported in 2014 (*3*). Those cases prompted a retrospective review of surveillance isolates that identified a case of *C. auris* bloodstream infection in 2009 at a Johannesburg academic hospital (*4*). The rapid emergence and international spread during 2014–2023, high crude mortality rates (30%–60%), and antifungal resistance make *C. auris* a global public health concern; the World Health Organization has designated it a critical-priority fungal pathogen (*5*,*6*).

Neonatal outbreaks caused by *C. auris* are occasionally documented, although early outbreaks in South Africa occurred mainly among critically ill adults (*7*). In more recent years, *C. auris* has caused outbreaks involving neonates and has subsequently become endemic in many hospitals in South Africa (*7*–*10*). *C. parapsilosis* has been a major pathogen causing late-onset sepsis among hospitalized neonates in low- and middle-income countries because of its ability to colonize the skin of hospitalized patients and healthcare workers, persist on hospital surfaces, and adhere to indwelling medical devices, characteristics that are also applicable to *C. auris* (*11*,*12*).

*C. auris* is classified into several genetically distinct clades. Clade I originated in South Asia, clade II in East Asia, clade III in Africa, clade IV in South America, and clade V in the Middle East (Iran) (*13*). All clades exhibit minimal intraclade genetic diversity but are separated from each other by tens to hundreds of thousands of single nucleotide polymorphisms (*13*,*14*). A possible sixth clade was reported from Singapore and Bangladesh in August 2023 (C. Suphavilai et al., unpub. data, https://www.medrxiv.org/content/10.1101/2023.08.01.23293435v1). Most clade isolates from Africa are resistant to fluconazole; some are resistant to other antifungal classes (*9*,*15*). Globally, echinocandin-resistant isolates are not as prevalent as azole- and polyene-resistant isolates, which is partly why echinocandins are recommended as first-line treatment (*5*). 

We aimed to describe the molecular epidemiology of *C. auris* at a large tertiary academic hospital in Johannesburg, South Africa by using whole-genome sequencing (WGS), focusing on a persistent outbreak in the hospital’s neonatal unit. For GERMS-SA, an ongoing surveillance program, we sought and obtained annual ethics approvals from several university ethics committees in South Africa. A detailed description of GERMS-SA surveillance methods has been published previously (*7*).

## Materials and Methods

### Study Setting and Definitions

We conducted the cross-sectional study at the 3,200-bed Chris Hani Baragwanath Academic Hospital (CHBAH) in Soweto, Johannesburg, South Africa. Affiliated with the University of the Witwatersrand, CHBAH provides a wide range of subspecialist inpatient and outpatient surgical, medical, pediatric and obstetrics/gynecology services. The hospital serves 1.5 million persons around Soweto and surrounding areas as a tertiary referral center. The hospital’s neonatal unit caters to neonates requiring admission from ≈20,000 annual births within the hospital; 3,000 births from local district hospitals and 10,000 births from the local clinics annually; and those requiring surgical services from the southern Gauteng and North West Provinces (*16*). All *C. auris* isolates in this study were received from the onsite microbiology laboratory at CHBAH. 

**Figure 1:**
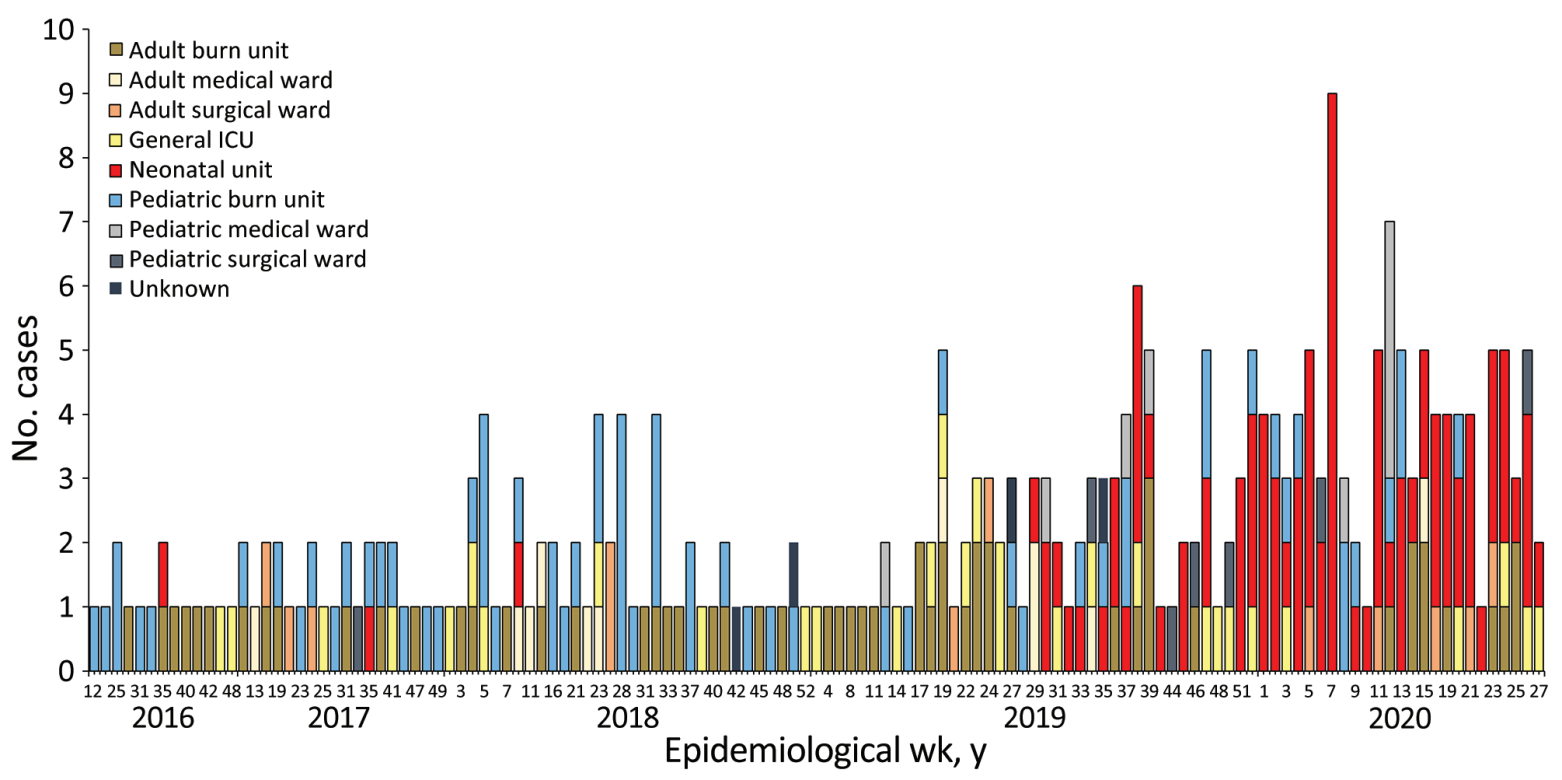
Epidemic curve by ward location for 287 laboratory-confirmed cases of *Candida auris* infection or colonization at an academic tertiary hospital, South Africa, March 2016–July 2020. ICU, intensive care unit.

We included *C. auris* isolates from all patients admitted to the neonatal unit from June 12, 2019, through May 30, 2020 (overlapping with the neonatal unit outbreak, which began with an index case diagnosed on June 12, 2019), as well as isolates from patients admitted across the hospital from March 1, 2016, through July 31, 2020 (Figure 1). Specimens that were processed for routine microbiology culture and yielded *C. auris* were from blood, central venous catheter (CVC) tips, urine, and wound and burn swabs. We also included *C. auris* isolates that were cultured from swabs collected by targeted screening of high-risk contact patients in the neonatal unit (e.g., adjacent incubator, same cubicle, or both). For all case-patients with multiple isolates within a 30-day period, we used the first isolate for analysis. We obtained demographic and clinical data such as age, sex, ward location, and site of *Candida* infection from the GERMS-SA surveillance database. We defined neonates as <28 days of age, infants as 28 days to <12 months of age, children as 1–12 years of age, adolescents as 13–17 years of age, and adults as >18 years of age.

### Culture, Identification, and Antifungal Susceptibility Testing

All isolates from invasive and noninvasive cases were first processed at the CHBAH onsite laboratory. If yeasts were observed on microscopic direct examination or if fungal culture was specifically requested, we inoculated specimens onto Sabouraud agar (Diagnostic Media Products [DMP], https://www.nhls.ac.za). We used Vitek-2 (bioMérieux, https://www.biomerieux.com) and Microscan Walkaway (Beckman Coulter, www.beckmancoulter.com) for initial identification. We shipped cultures to the National Institute for Communicable Diseases (NICD) on Dorset transport medium or agar plates (DMP); we confirmed identification and performed antifungal susceptibility testing before storage (*16*,*17*). For this study, we retrieved *C. auris* isolates from −70°C freezer storage at the NICD and subcultured on Sabouraud agar to confirm viability, then cultured them onto chromogenic agar (DMP) to confirm purity (CHROMagar *Candida* powder was sourced from Mast Diagnostics, https://www.mast-group.com). We conducted matrix-assisted laser desorption/ionization-time of flight mass spectrometry (Bruker Daltonics, https://www.bruker.com) to identify all *C. auris* isolates again before further testing. We performed antifungal susceptibility testing using commercial gradient diffusion (Etest, bioMérieux) or broth microdilution assays (Sensititre; Thermo Fisher, https://www.thermofisher.com). We used the Etest method to generate amphotericin B MICs because the gradient diffusion strip provides a wider range of MIC values than are generated by broth-microdilution testing; the wider range may help discriminate between susceptible and resistant isolates (*17*). We interpreted MICs using tentative breakpoints proposed by the US Centers for Disease Control and Prevention (*1*). All experiments included the quality control strains *C. parapsilosis* (ATCC 22019) and *C. krusei* (ATCC 6258), following M27-A3 (*18*) and M60 recommendations of the Clinical and Laboratory Standards Institute (*19*).

### Whole-Genome Sequencing

We extracted DNA using the Zymo Research Quick-DNA Fungal/Bacterial miniprep kit (Zymo Research Corporation, https://www.zymoresearch.com) according to the manufacturer’s instructions, with some exceptions. For example, we extracted DNA from yeast colonies directly instead of from a resuspension of yeast cells in water; we used 40 µL of elution buffer instead of the 100 µL recommended volume. We sent genomic DNA to the NICD Sequencing Core Facility for WGS. We performed sequencing of the 12.6 Mb-sized genome on 188 samples. We prepared genomic libraries using Nextera DNA Library Preparation (Illumina, https://www.illumina.com), followed by 2 × 300 bp sequencing on an Illumina NextSeq 500 platform to produce paired end reads.

### Single-Nucleotide Polymorphism Calling and Phylogenetic Analysis

We assessed the quality of read data using FastQC version 0.11.9 (https://www.bioinformatics.babraham.ac.uk) and performed read filtering and trimming using PRINSEQ version 0.20.4 (http://prinseq.sourceforge.net) (*20*). We mapped the read data to a reference *C. auris* genome (clade III, B11221; National Center for Biotechnology Information [NCBI] BioProject accession no. PRJNA535510) using the Burrows-Wheeler data transformation algorithm version 0.7.17 (*21*). We performed variant calling (i.e., single-nucleotide polymorphism [SNP] analysis) using SAMtools version 1.11 (*21*) through the Northern Arizona SNP Pipeline (NASP) (http://tgennorth.github.io/NASP/install.html) (*22*). Filtering parameters involved removing positions that had <10× coverage, <90% variant allele calls and those within duplicated regions in the reference (*14*). We estimated a maximum parsimony phylogeny tree using MEGA software version 10.0.5 (https://www.megasoftware.net) and bootstrap analysis based on 1,000 replicates using the bestSNP alignment produced by the NASP pipeline (*23*). We included external sequences representing each clade (I–V) in the analysis to confirm the clade assignments of the isolates: clade I, PEKT02 (B8441, NCBI BioSample no. SAMN05379624) and SRR3883445 (B11214, NCBI BioSample no. SAMN05379602); clade II, PYFR01 (B11220, NCBI BioSample no. SAMN05379608); clade IV, PYGM01 (B11243, NCBI BioSample no. SAMN05379619); clade V, SRR9007776 (NCBI BioSample no. SAMN11570381). Also included in the analysis was a genome of the first South Africa isolate to be reported and sequenced from CHBAH in 2009 (*9*).

### Phylodynamic Analysis of Clade III Neonatal Isolates

We created a root-to-tip regression plot using TempEst version 1.5.3 (*24*) to quantify the mutation rate and to assess the temporal signal of the outbreak strains. We inferred Bayesian phylogenies using BEAST version 1.8.4 (*25*) by applying a general time-reversible nucleotide substitution model under a strict molecular clock with the mutational rate obtained from the root-to-tip regression. We used the SNP alignment of the clade III isolates for both TempEst and BEAST. We chose a general time-reversible model as a nonspecific model for phylogenetic inference. The model assumes different rates of substitution for each nucleotide and different frequencies of nucleotide occurrence (*26**)*. Furthermore, we applied a coalescent exponential population prior distribution and used specimen collection dates (day, month, year) as tip dates. We set the length of the Markov Chain Monte Carlo (MCMC) at 50,000,000 steps and logged parameters every 1,000 steps. We used Tracer version 1.7.1 (https://beast.community/tracer) to visualize and analyze the posterior MCMC samples. The effective sample size was >700, indicating that the MCMC runs had converged. We generated a maximum clade credibility tree with TreeAnnotator version 1.8.4 (https://beast.community/treeannotator) after discarding the first 10% as burn-in and visualized the tree using FigTree version 1.4.4 (http://tree.bio.ed.ac.uk/software/figtree). We also performed an exploratory analysis to reconstruct potential transmission routes in the hospital (Appendix).

### Resistance Mutation Identification

We used CLC Genomics Workbench version 10 (QIAGEN, https://www.qiagen.com) to assess and identify mutations in resistant *C. auris* isolates. We extracted 2 genes from each resistant *C. auris* genome and assessed for any point mutations (i.e., substitutions, duplications): *ERG11*, which is transcribed into an azole target; and *FKS1*, an echinocandin target gene (*27*). We used a wild-type strain with no known mutations as a reference in the analysis to determine the presence of mutations (*C. auris* B8441, NCBI accession no. PEKT00000000). Susceptible isolates were also assessed for mutations in the same genes.

## Results

### Patient Characteristics

During the surveillance period, 287 culture-confirmed cases (invasive and noninvasive) were reported from the hospital from different wards; of those, 207 (72%) had viable isolates available for analysis. Most of the cases were from the neonatal unit (91/287, 32%), pediatric burn unit (66/287, 23%), and adult burn unit (57/287, 20%). The median age was 1.4 years (interquartile range [IQR] 22 days to 21 years; range 0 days to 85 years). Adults accounted for the highest proportion of cases (74/287, 26%) followed by neonates (62/287, 22%). More patients were male (54%, 155/287) than female (42%, 121/287; sex was unknown for 4% (11/287). Most isolates were from blood cultures (161/287, 56%), followed by skin swabs (33/287, 12%) (Table 1).

**Table 1:** Characteristics of 287 patients with *Candida auris* infection or colonization admitted to a large academic hospital in South Africa, 2016–2020

Characteristic	No. (%) isolates
Sex	
M	155 (54)
F	121 (42.2)
Unknown	11 (3.8)
Age group	
Neonates, <28 d	62 (21.6)
Infants, 28 d–12 mo	56 (19.5)
Children, 1–12 y	54 (18.8)
Adolescents, 13–17 y	11 (3.8)
Adults, >18 y	74 (25.8)
Unknown	30 (10.5)
Specimen type	
Blood	161 (56.1)
Skin swab	33 (11.5)
Arterial catheter tip	28 (9.8)
Central venous catheter tip	24 (8.4)
Urine	11 (3.8)
Tracheal aspirate	8 (2.8)
Tissue, not specified	6 (2.1)
Fluid aspirate, not specified	3 (1)
Skin scraping	1 (0.3)
Burn/wound swab	1 (0.3)
Unknown	11 (3.5)
Ward location	
Neonatal unit	91 (31.7)
Pediatric burn unit	66 (23)
Adult burn unit	57 (19.9)
General adult/pediatric intensive care unit	30 (10.5)
Adult surgical	12 (4.2)
Adult medical	11 (3.8)
Pediatric medical	9 (3.1)
Pediatric surgical	7 (2.4)
Unknown	4 (1.4)

### Antifungal Susceptibility of Isolates

Most (199/207; 96%) of the isolates were resistant to fluconazole (MIC >32 µg/mL); 13 (6%) were considered resistant to amphotericin B (MIC >2 µg/mL). Multidrug-resistant isolates were also present; 2 (0.9%) isolates were resistant to both caspofungin and fluconazole and 3 (1.4%) were resistant to both fluconazole and amphotericin B. No isolates in this study were resistant to >3 antifungal classes (panresistant) (Table 2).

**Table 2:** MIC distribution of *Candida auris* isolates (n = 207) from patients admitted to a tertiary academic hospital, South Africa, 2016–2020*

Drug	Breakpoint, µg/mL	No. isolates with MIC, µg/mL																Unk†	% Resistant
		0.008	0.015	0.03	0.06	0.12	0.25	0.5	1	2	4	8	16	32	64	128	256		
AFG	≥4		1		28	129	42	7											0
MFG	≥4			2	96	94	12	1	2										0
5FC	≥128	3			67	125	10	2											0
POS	NA	1	1	15	73	80	31	4	2										NA
VRC	NA				2	8	21	58	103	13	2								NA
ITC	NA			2	10	84	84	25	2										NA
FLC	≥32								1				7	13	34	73	79		96
AMB	≥2						1	9	135	61								1	42
AMB Etest‡	≥2		1	1	2	9	22	76	79	11	2							4	6.2

*AMB, amphotericin B; AFG, anidulafungin; FLC, fluconazole; ITC, itraconazole; MFG, micafungin; NA, not available; POS, posaconazole; unk, unknown; VRC, voriconazole; 5FC, flucytosine. †Isolates missing data or not tested ‡MICs were determined using both broth microdilution and E-test method for amphotericin B.

### Phylogenetic and Resistant Mutation Analysis

**Figure 2:**
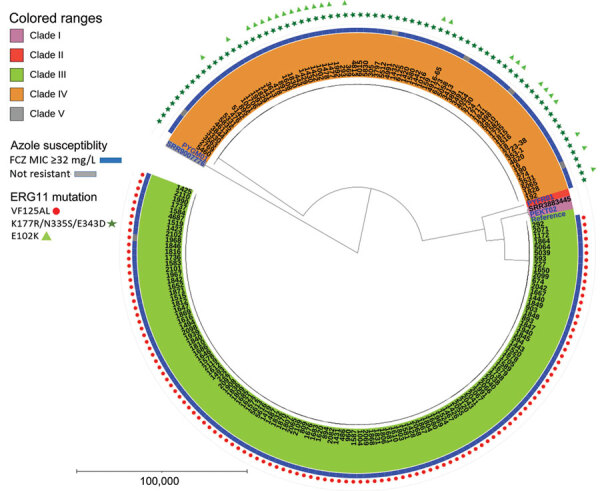
Phylogenetic tree depicting clade distribution and fluconazole resistance mutations of 188 invasive or colonizing South African *Candida auris* strains isolated from patients admitted to a large metropolitan hospital in South Africa, 2016–2020. The unrooted maximum-parsimony tree was created using MEGA software (https://www.megasoftware.net) using 287,338 single-nucleotide polymorphisms based on 1,000 bootstrap replicates. Scale bar indicates number of pairwise differences. FCZ, fluconazole.

Of 207 viable *C. auris* isolates, 200 were available for WGS. We excluded 12 sequences of poor quality from the analysis; WGS data of 188 isolates were available for bioinformatics analysis. Overall, 118 (63%) clustered with the Africa clade III reference and the remaining 70 (37%) with the South America clade IV reference (Figure 2). Isolates in this study did not cluster in any other clade. A total of 181 (96%) isolates had a fluconazole MIC value >32 µg/mL, and 186 (99%) had known resistance mutations in the *ERG11* gene; thus, 5 sequences from phenotypically susceptible isolates also housed resistance mutations (Table 3). Isolates for clade III had the VF125AL substitution that is specific to the clade. Isolates from clade IV had the substitutions K177R/N335S/E343D, which had been documented in this clade previously *(**28**)*. Some isolates with the clade IV substitutions (27/70, 38%) had an additional E102K substitution. 

**Table 3:** Clade proportions and frequency of antifungal drug resistance within the study population among 188 *Candida auris* isolates, South Africa

Clade	No. (%) isolates				
	All	Fluconazole resistant	Point mutation in *ERG11*	Point mutation in *FKS1*	Amphotericin B resistant
III	118 (62.5)	117 (99.1)	118 (100)	0	2 (2.8)
IV	70 (37.4)	66 (91.4)	70 (100)	0	1 (1.42)

**Figure 3:**
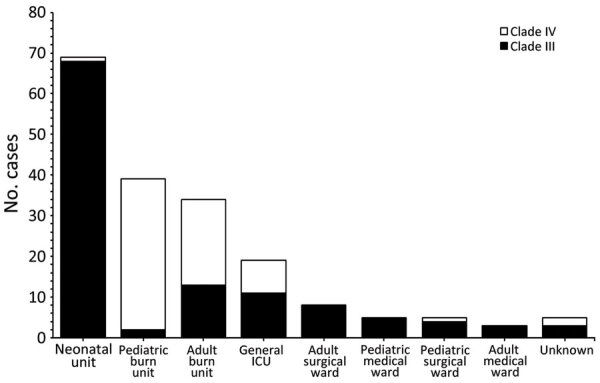
Clade distribution of 188 South African *Candida auris* isolates from patients admitted to a large metropolitan hospital classified by the patients’ ward locations, South Africa, 2016–2020. ICU, intensive care unit.

The neonatal unit, the location of the outbreak starting in June 2019, was dominated by clade III isolates, whereas clade IV strains dominated the adult and pediatric burn units. We observed clade mixing in both burn units (Figure 3). Isolates from the neonatal unit constituted 37% (68/188) of the isolates in our phylogenetic tree.

### Phylodynamic Analysis

**Figure 4:**
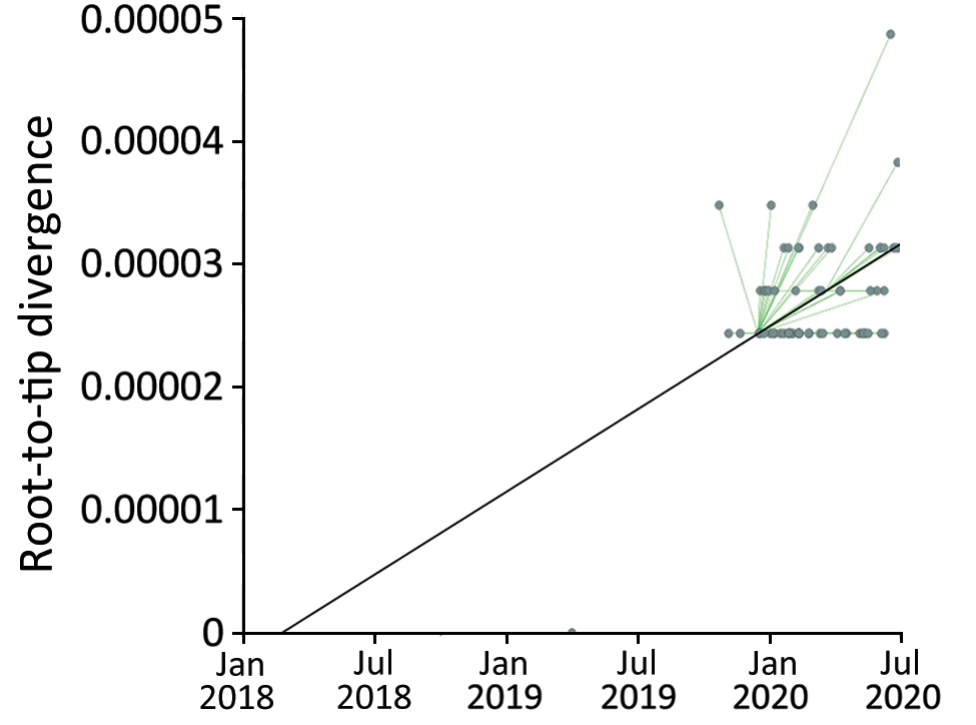
Root-to-tip regression analysis of 67 South African *Candida auris* outbreak isolates from the neonatal ward of a large metropolitan hospital in South Africa. Genetic distance is plotted against sampling time. Every data point represents a tip on the phylogeny. Black line indicates correlation coefficient for the regression. Green lines represent the evolutionary rate in substitutions/site/day.

**Figure 5:**
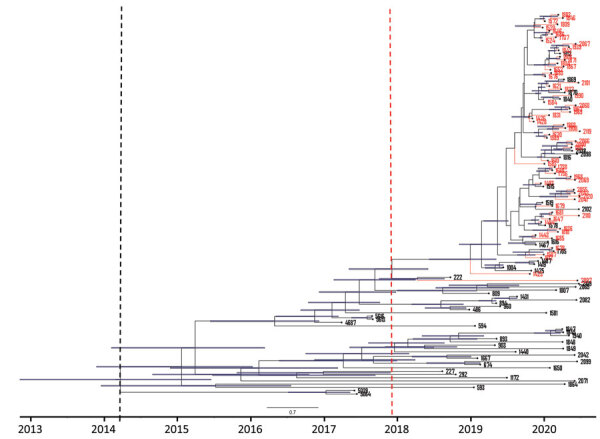
Maximum clade credibility tree of 118 South Africa clade III *Candida auris* isolates from patients at an academic tertiary hospital in South Africa estimated using BEAST strict clock and coalescent model (*24*). Red tips represent cases from the neonatal ward, blue bars represent 95% highest probability density black dashed line indicates clade III tMRCA, and red dashed line indicates outbreak strain tMRCA. tMRCA, time to most recent common ancestor.

We included only isolates from clade III, which was the clade responsible for the neonatal unit outbreak, in the following analysis. We created a root-to-tip regression plot to determine whether there was a positive correlation between the time the isolates were sampled and the number of substitutions along the tree topology. Linear regression estimates of the evolutionary rate equated to 1.3471 × 10^5^ nt substitutions/site/year (correlation coefficient = 0.5). The rate was similar to that of an outbreak in Kenya caused by clade III isolates in which the calculated mutation of the outbreak strains was 1.8695 e-5 substitutions/site/year (*29*). The rate supported a strong temporal signal and was sufficient for further Bayesian molecular dating. The root-to-tip regression plot predicted the time to the most recent common ancestor (tMRCA) in 2018 based on the x intercept (Figure 4). The tMRCA for clade III in this hospital dated back to early 2014 (95% highest posterior density 2013 to mid-2015), whereas the tMRCA for the neonatal unit outbreak dated to 2018 (95% highest posterior density, mid-2017 to mid-2018), corresponding to the estimation from the root-to-tip regression analysis (Figure 5). The exploratory analysis of the outbreak reconstruction of clade III revealed that infections were introduced into the neonatal unit from the adult burn unit, adult medical department, and an unknown ward (Appendix).

## Discussion

Fluconazole-resistant clade III *C. auris* isolates caused a large persistent outbreak in the neonatal unit of a tertiary academic South Africa hospital beginning in June 2019 and continuing into 2023. WGS analysis of all viable surveillance isolates from this hospital during 2016–2020 revealed that clade IV *C. auris* isolates dominated in the adult and pediatric burn units with clade III and IV co-circulation. Phylodynamic analysis of the clade III neonatal outbreak isolates did not point to a specific source.

By 2016, *C. auris* was reported in >100 South Africa acute-care hospitals and had caused large outbreaks in some of these hospitals. *C. auris* had replaced other pathogens to become the third most common cause of candidemia in South Africa (*7*). Over our entire study period (i.e., March 2016–July 2020), the neonatal unit outbreak contributed to one third of all the cases at the hospital. Smaller *C. auris* outbreaks involving neonates have been reported previously in other hospitals in South Africa (*7*,*30*). Neonatal units in national central hospitals, such as CHBAH, have become fungal outbreak epicenters, driven by the innate vulnerability of very high-risk patients admitted to these units, occupancy routinely exceeding the approved bed capacity of the unit, and limited resources available for infection prevention and control or antimicrobial stewardship (*31*). Although the exact manner of *C. auris* introduction for this outbreak remains undetermined, temporal analysis of the outbreak strains placed tMRCA in early 2018, suggesting a relatively recent introduction (*10*,*32*). Continued surveillance until June 2022 has confirmed persistence of *C. auris* in the unit with sporadic cases of invasive infections.

Most isolates in our study belonged to Africa clade III (118/188, 63%) or the South America clade IV (70/188, 37%). The overall molecular epidemiology of *C. auris* in South Africa was determined in a WGS study which reported that 85% of isolates from 2009–2018 belonged to clade III, 12% belonged to clade I, and 3% to clade IV (*9*). An earlier global molecular epidemiology study reported only clade III isolates (n = 51) from South Africa (*33*). However, clade IV contributed to a proportionally larger case load in this hospital. Because the first reported isolate in South Africa in 2009 belonged to clade IV and was from CHBAH (*9*), we hypothesize that a clonal expansion of clade IV occurred in this hospital and those strains continued to circulate during our study period. Circulation of multiple *C. auris* clades in South Africa may be explained by global travel and trade (*34*–*36*). Furthermore, phylogeographic mixing of *C. auris* clades has been observed more often in recent years. Countries including Canada (clades I, II, and III), Kenya (clades I and III) and the United States (clades I, II, III, and IV) also reported cases caused by multiple *C. auris* clades (*10*).

Most isolates in our study were resistant to fluconazole. Fluconazole-susceptible isolates of *C. auris* are rare worldwide, especially within Africa clade III; this resistance limits the treatment options (*9*). Fluconazole-resistant *C. auris,* as a cause of healthcare-associated infections, adds costs to the health system since fluconazole is the least expensive and most accessible systemic antifungal agent (*8*,*37*). Amphotericin B resistance has been reported among *C. auris* isolates but remains rare while echinocandin resistance has also been reported, especially in clade I isolates (*37*). Echinocandins are recommended as first-line treatment for candidemia in adults, although amphotericin B deoxycholate, with better central nervous system penetration, is recommended for neonates who may develop *Candida* meningoencephalitis (*8*). We did not observe isolates resistant to all classes of antifungals in this study; however, 2 panresistant isolates were reported in South Africa in a previous study (*16*).

Most of the fluconazole-resistant isolates in our study had clade-specific mutations in the *ERG11* gene that are known to contribute to resistance. The clade III *ERG11* mutation (VF125AL) seems to be universal across resistant isolates within the clade and has been seen in other clade III isolates from previous studies, even those isolated outside Africa (*33*; T.K. Price et al., unpub. data, https://doi.org/10.1101/2020.10.26.20214908). The *ERG11* gene–mediated mechanism of resistance to fluconazole for clade IV isolates varies, and isolates from different geographic areas have different *ERG11* mutations (*28*)*.* Isolates in our study had the same substitutions as isolates originating from Colombia, which contained all 3 Erg11p substitutions (K177R, N335S, E343D), whereas azole resistance in clade I India/Pakistan isolates and some clade IV Colombia isolates was caused by Y132F or K143R substitutions (*28*). Of interest, the K177R, N335S, and E343D clade IV substitutions have not been shown to contribute to increased azole resistance even in Colombian isolates. Instead, other Colombian isolates had the substitutions I466M or Y501H that have been shown to contribute to resistance, which we did not see in the isolates in our study (*28*). Some sequences with the described clade IV substitutions had an additional E102K substitution in our study; this substitution has not been documented or proven to contribute to decreased susceptibility in *C. auris* strains. There were cases in our study where susceptible isolates possessed resistant mutations especially in clade IV. It appears that clade IV isolates might have a more complex mechanism of resistance.

A limitation of our study was that we only included cultured isolates from laboratory-based surveillance from patients with routinely diagnosed invasive infections and a few with colonization. Thus, we sampled only a subset of all infections that would have occurred at this hospital. An extensive collection of isolates using systematic sampling in a prospective study, including invasive, colonizing, and environmental strains, would be needed to determine the full extent of transmission routes with a higher resolution and to avoid missing links. In addition, detailed clinical information and travel and hospital transfer data were not available for all cases; this information would have enabled us to potentially determine how the pathogen was introduced into the hospital. Another limitation in our study is that we did not account for recombination analysis, which might have introduced bias in the phylogenetic analysis.

In summary, we characterized *C. auris* isolates circulating in a major metropolitan public hospital in South Africa. Our data showed that clades III and IV co-circulated, and clade III was responsible for a large outbreak in the neonatal unit. Most isolates were resistant to fluconazole and carried previously published clade-specific *ERG11* mutations. We speculate that the neonatal unit outbreak may have originated from cross-unit transmission by infected or colonized patients, colonized healthcare workers, or contaminated equipment. Patient environments may have also served as reservoirs of infection.

Appendix Additional information for study of *Candida auris* clinical isolates associated with outbreak in neonatal unit at tertiary academic hospital, South Africa.
