# Supplementary material for: Candida auris Clinical Isolates Associated with Outbreak in Neonatal Unit of Tertiary Academic Hospital, South Africa
Source: Emerg Infect Dis. 2023 Oct;29(10):2044–53. doi: 10.3201/eid2910.230181 (PMC10521600; doi:10.3201/eid2910.230181)
Supplement: Appendix — Additional information for study of Candida auris clinical isolates associated with outbreak in neonatal unit at tertiary academic hospital, South Africa. [file 23-0181-Techapp-s1.pdf]

Article DOI: http://doi.org/10.3201/eid2910.230181

EID cannot ensure accessibility for supplementary materials supplied by authors. Readers who have difficulty accessing supplementary content should contact the authors for assistance.

Candida auris Clinical Isolates Associated with Outbreak in Neonatal Unit of Tertiary Academic Hospital, South Africa

Appendix

Supplementary Methods

Transmission Route Reconstruction

To reconstruct potential transmission routes in the hospital, an algorithm called SeqTrack was used (*1*). SeqTrack is within the adegenet package in R, it uses a parsimony phylogeny approach to determine ancestries based on an alignment of SNPs and sampling dates of the isolates to create a schematic diagram of proposed transmission routes between the patients. The mutational rate (mu) obtained from the root-to-tip regression analysis, the length (haplo.length) of analyzed sequences in number of nucleotides and a matrix of pairwise distances and collections was used to plot a series of trees and the tree with the maximum parsimony was chosen. Only one sample per person (whether colonization or disease) was used. The data from the isolates was assessed to investigate whether the isolates formed distinct clusters due to epidemiologic variables such as year of isolation or ward location with regard to their genetic relationships.

Supplementary Results

Outbreak Reconstruction for Clade III

The potential infection chains or transmission route of *C. auris* clade in the hospital were determined using SeqTrack. The transmission network shows the most likely source of infection/colonization for each case. Only isolates from clade III were included in this analysis, which was the clade responsible for the outbreak in the neonatal ward. This analysis allowed us

to track clade III strains across the hospital wards and introduction into the neonatal ward (Appendix Figure).

Case 1 (Isolate 4867 on the phylogenetic trees) was the index case of this tree and was isolated from a patient in an adult medical ward. Two transmission events occurred from Case 1 that gave rise to two clusters. The first cluster begins with Case 37 (1172) from an unknown ward. This cluster contained 18 cases with an average of 12.2 pairwise differences and therefore most (n = 6) had <12 SNPs in this cluster and likely had a very recent common ancestor and transmission. The second cluster from Case 1 begins with Case 6 (227) from the adult burn unit. This cluster contained infections from the neonatal ward outbreak with all (n = 83) cases having <12 SNPs and an average of 1.3 pairwise differences. Case 18 (594) seems to be the index case/ancestral case for most infections involved in this outbreak. From this ancestor, the weight is as low as zero in most branches, which indicates rapid transmission of the pathogen between the cases, a characteristic of an outbreak. This is contrary to the high number of mutations between the cases earlier in the transmission tree; these cases were sporadic and are further apart in their sampling dates. Overall, infections appear to have been introduced into the neonatal unit from the adult burn unit, adult medical department and an unknown ward.

The Bayesian analysis using BEAST estimated that the TMRCA of the outbreak strain occurred in 2018. This estimation was supported by the date of isolation for Case 6 (227) and Case 10 (292), ancestors of the neonatal ward outbreak, which were both isolated in 2018.

This exploratory analysis is limited by the inclusion of only one sample per patient and that only a small subset of isolates from the hospital were collected which does not capture the full scope of transmission in the hospital. Furthermore, our study does not include environmental isolates which form a crucial part in the transmission of the pathogen and therefore introducing missing links in the network and a looser interpretation.

References

1. Jombart T, Eggo RM, Dodd PJ, Balloux F. Reconstructing disease outbreaks from genetic data: a graph approach. Heredity. 2011;106:383–90. PubMed https://doi.org/10.1038/hdy.2010.78

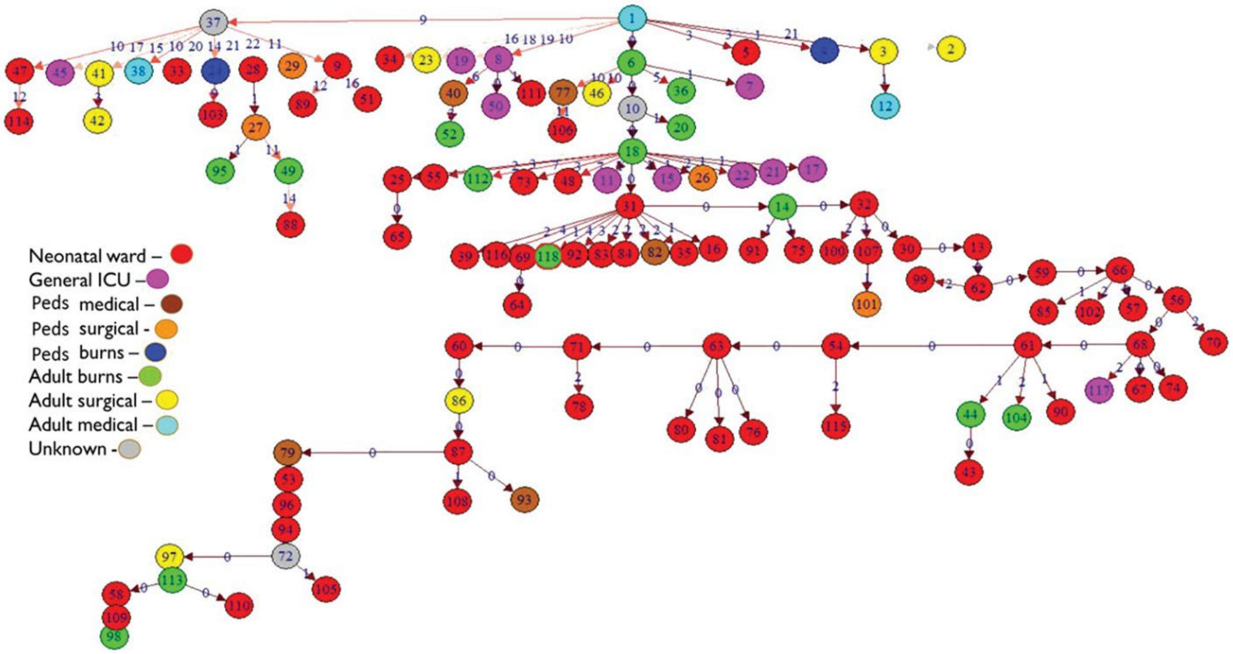

Appendix Figure. Reconstructed transmission tree of 118 South African Clade III Candida auris isolates from this study. Each case is represented by a node on the tree and arrows indicate plausible ancestries/transmissions. Mutations between the nodes are indicated by the color arrows (red = no/few mutations; light gray = many mutations) and the numbers in blue represent the weight of mutations when compared to the ancestries. Time is represented by y-axis (up: older; down: younger).
